# Supplementary material for: Structural Characterization and Anti-Osteoporosis Effects of a Novel Sialoglycopeptide from Tuna Eggs
Source: Mar Drugs. 2023 Oct 31;21(11):573. doi: 10.3390/md21110573 (PMC10672638; doi:10.3390/md21110573)
Supplement: Supplementary file 1 [file marinedrugs-21-00573-s001.zip › marinedrugs-2660596-supplementary.pdf]

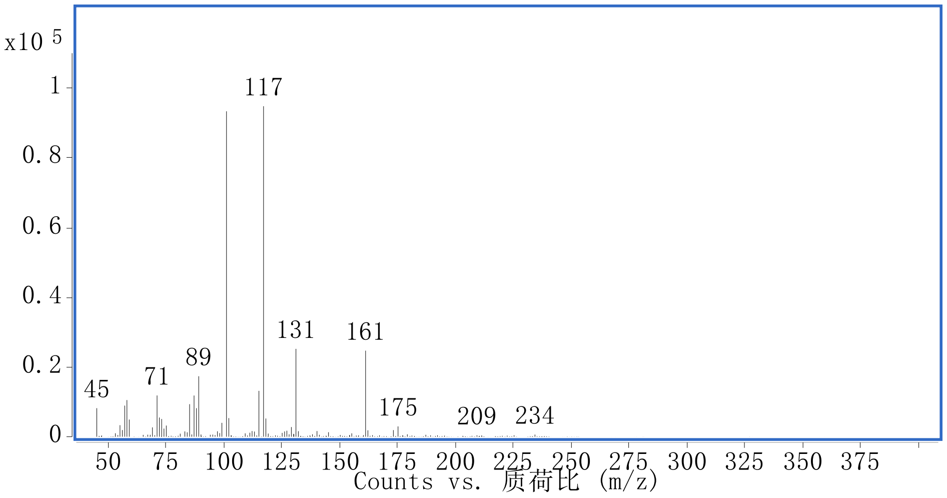

Supplementary Fig. S1 The mass spectra and proposed fragmentation 2,3,4-Me_3-Fuc

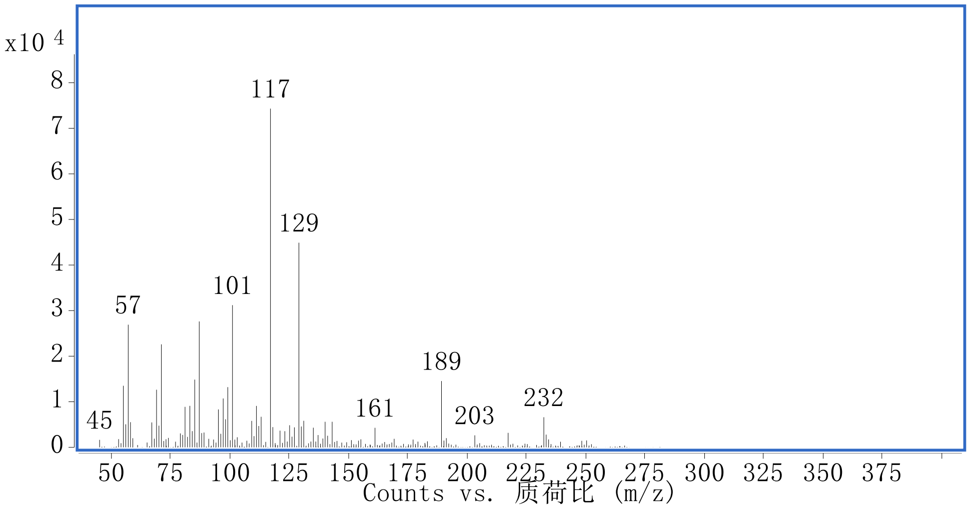

Supplementary Fig. S2 The mass spectra and proposed fragmentation 2,3-Me_2-Xyl

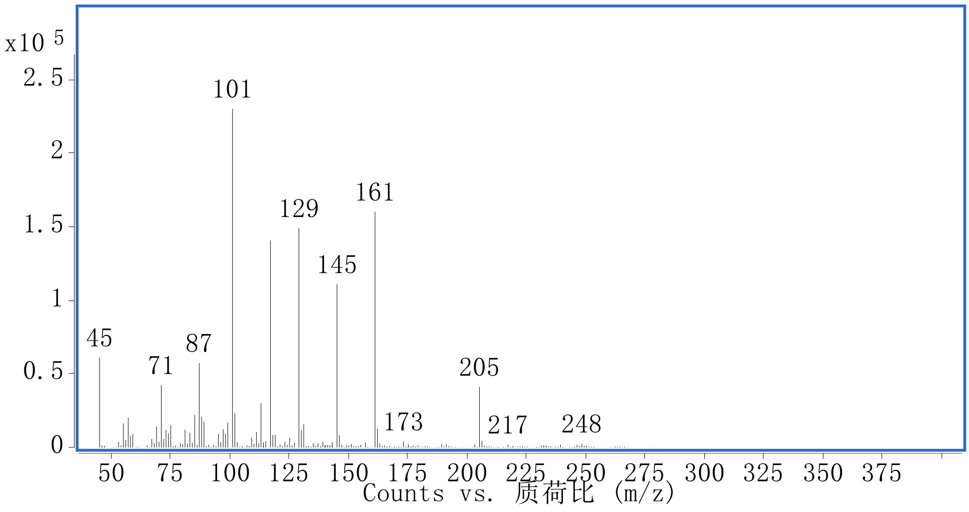

Supplementary Fig. S3 The mass spectra and proposed fragmentation 2,3,4,6-Me_4-Glc

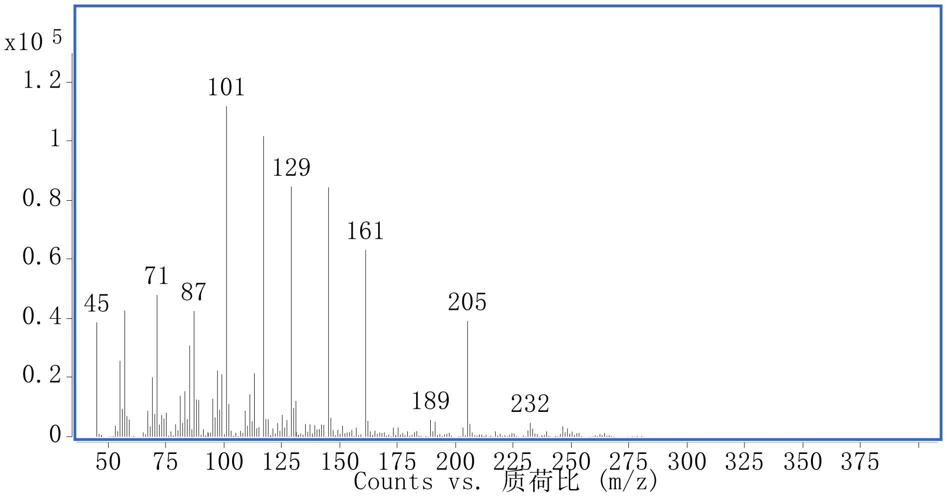

Supplementary Fig. S4 The mass spectra and proposed fragmentation 2,3,4,6-Me_4-Man

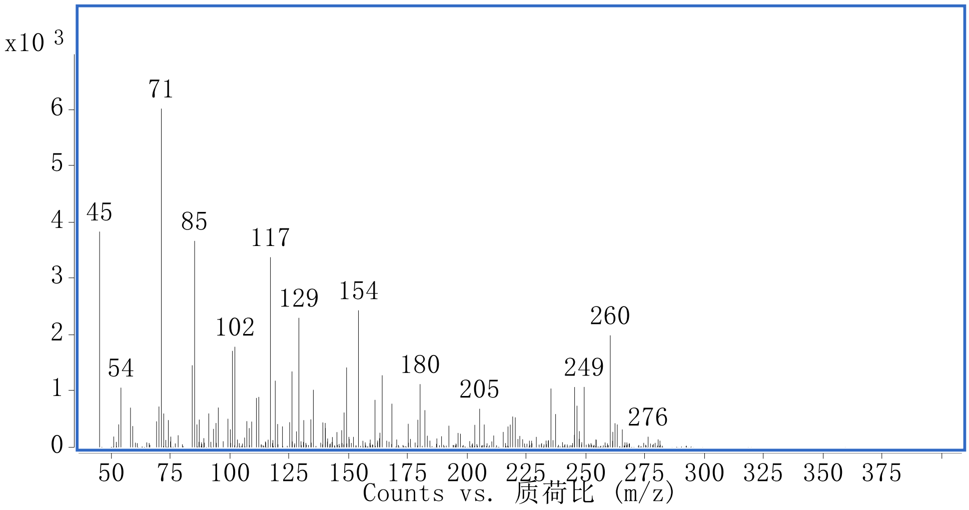

Supplementary Fig. S5 The mass spectra and proposed fragmentation 3,4,6-Me_3-D-Glc-2-N

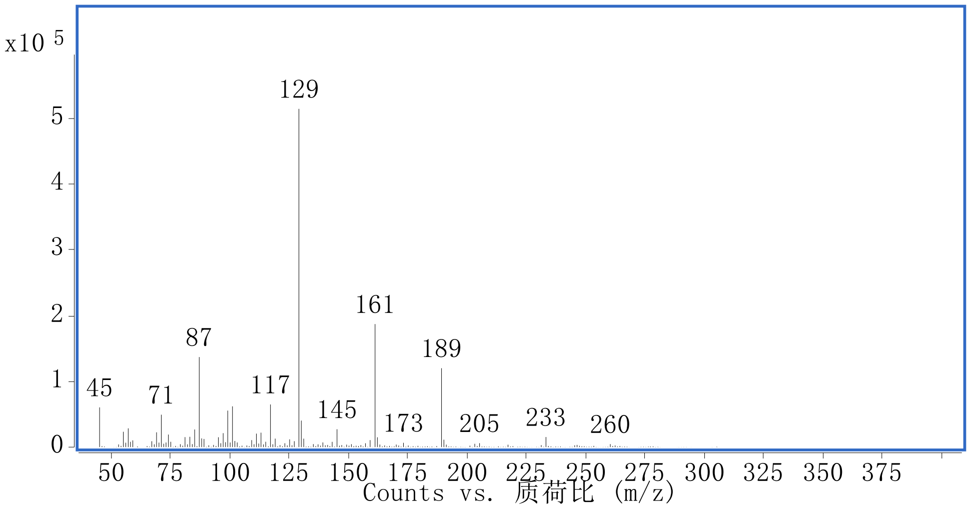

Supplementary Fig. S6 The mass spectra and proposed fragmentation 3,4,6-Me_3-Man

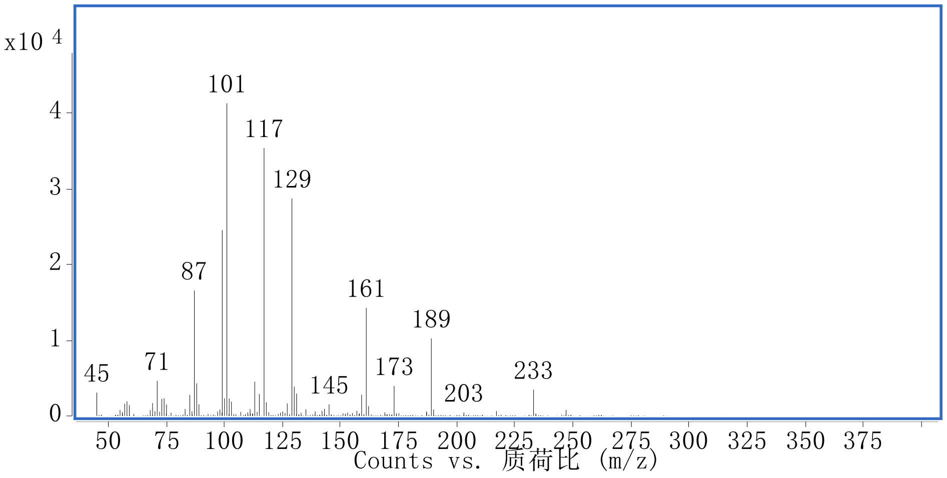

Supplementary Fig. S7 The mass spectra and proposed fragmentation 2,4,6-Me3-Glc

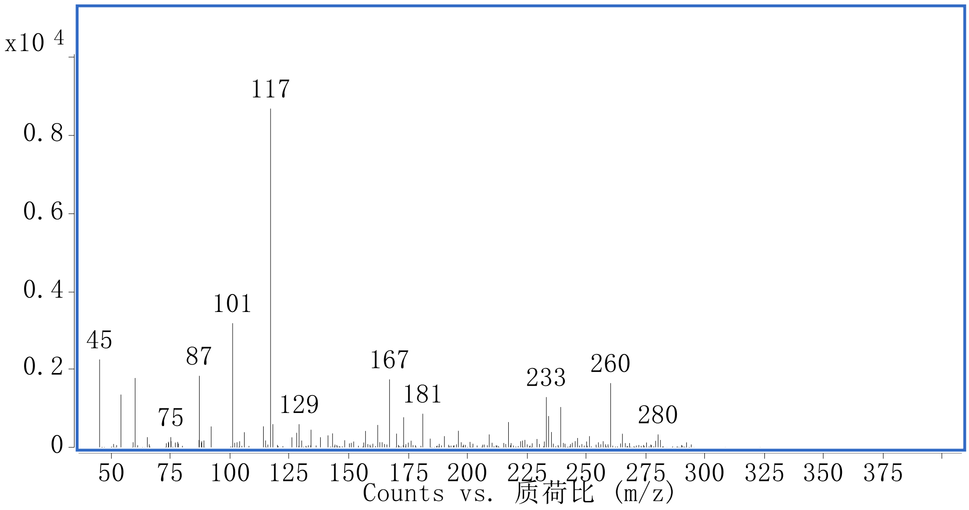

Supplementary Fig. S8 The mass spectra and proposed fragmentation 2,3,6-Me3-Glc

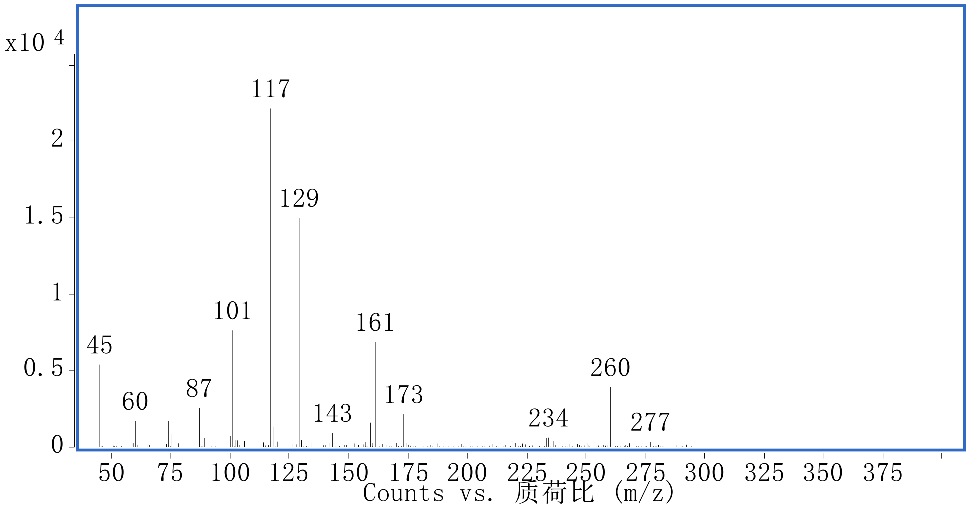

Supplementary Fig. S9 The mass spectra and proposed fragmentation 2,4,6-Me3-Gal

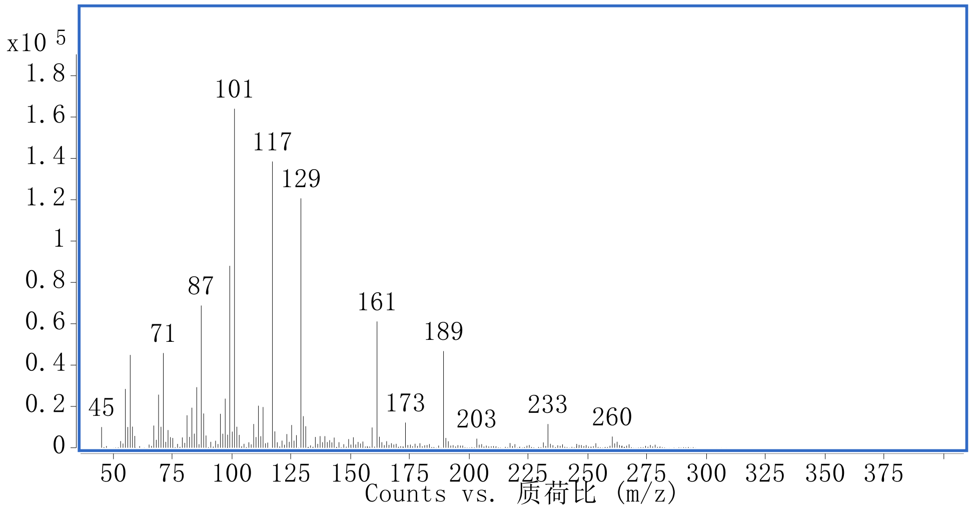

Supplementary Fig. S10 The mass spectra and proposed fragmentation 2,3,4-Me_3-Glc

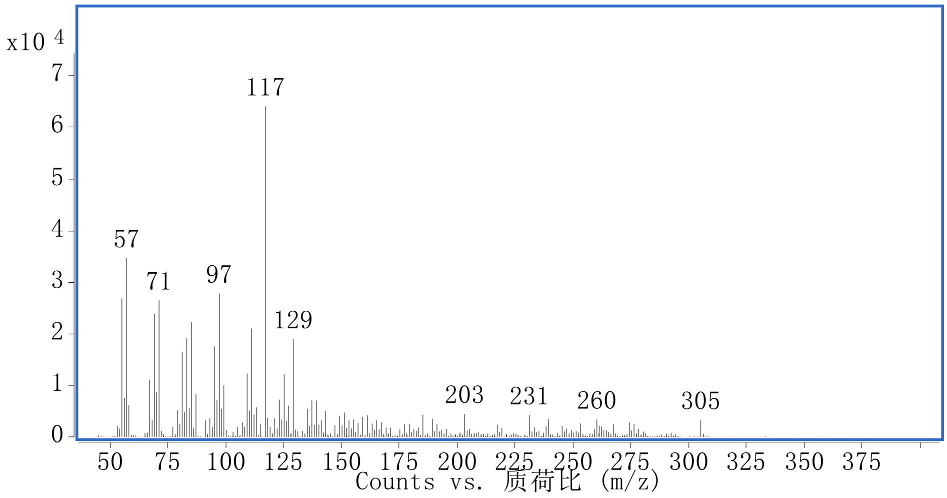

Supplementary Fig. S11 The mass spectra and proposed fragmentation 2,6-Me_2-Glc

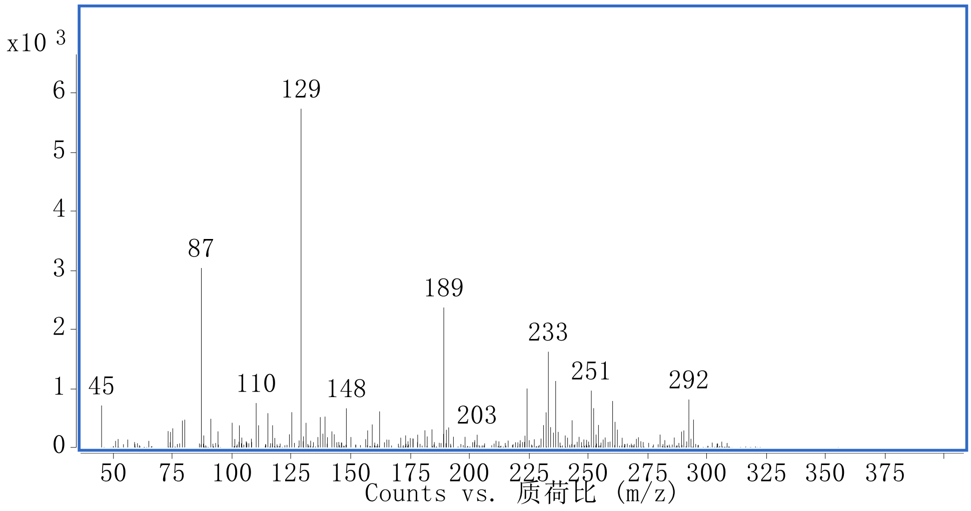

Supplementary Fig. S12 The mass spectra and proposed fragmentation 2,3-Me_2-Man

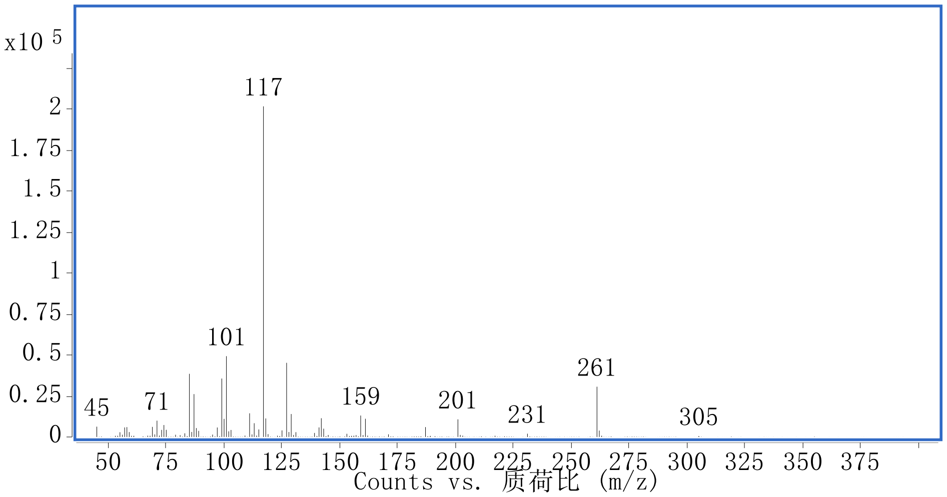

Supplementary Fig. S13 The mass spectra and proposed fragmentation 2,3-Me2-Glc

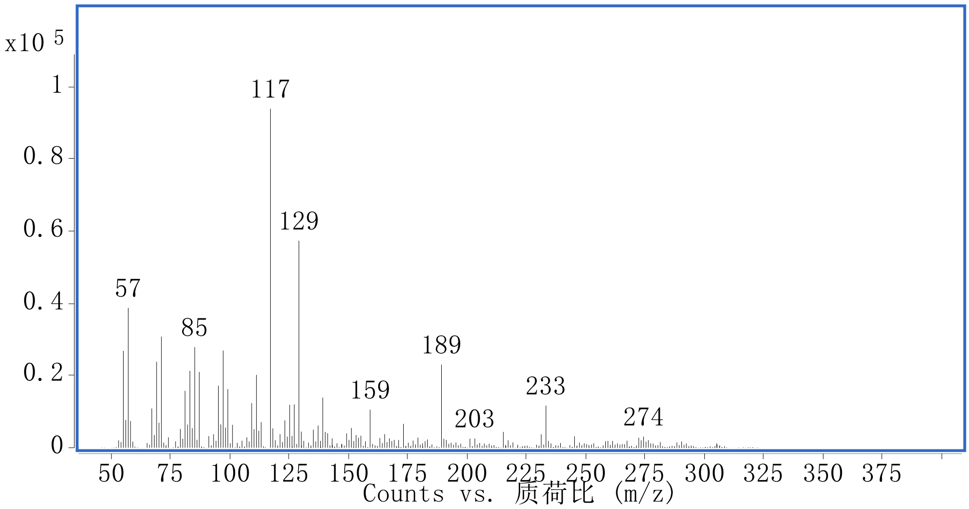

Supplementary Fig. S14 The mass spectra and proposed fragmentation 2,4-Me2-Glc

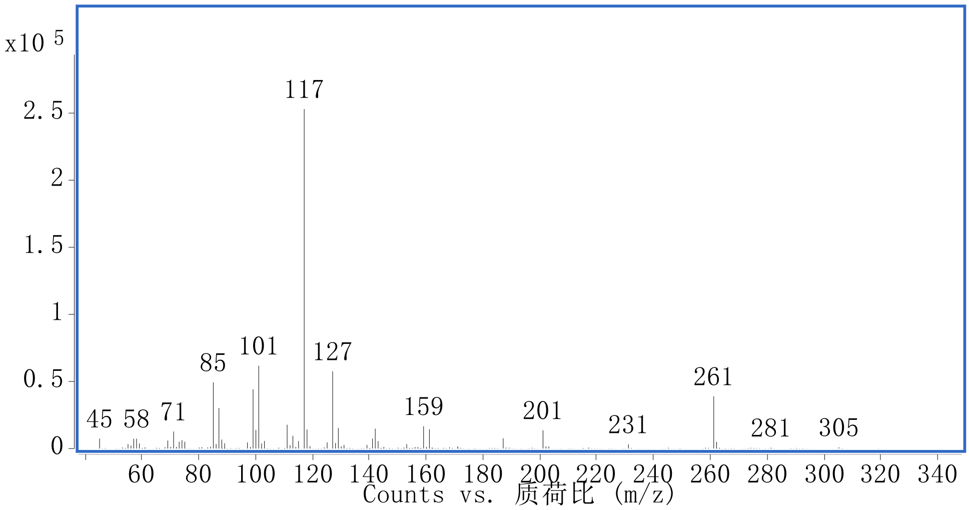

Supplementary Fig. S15 The mass spectra and proposed fragmentation 2,3-Me2-Gal

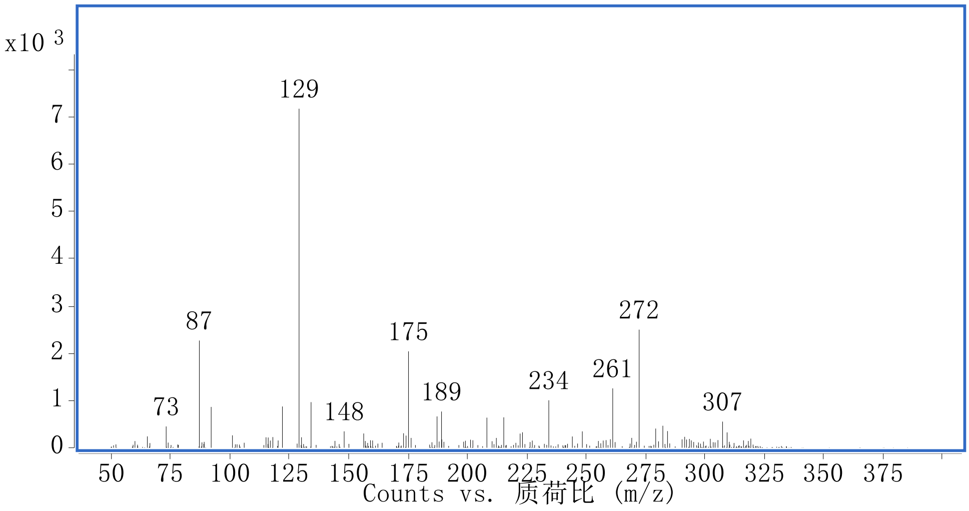

Supplementary Fig. S16 The mass spectra and proposed fragmentation 4,6-Me_2-D-Gal-2-N

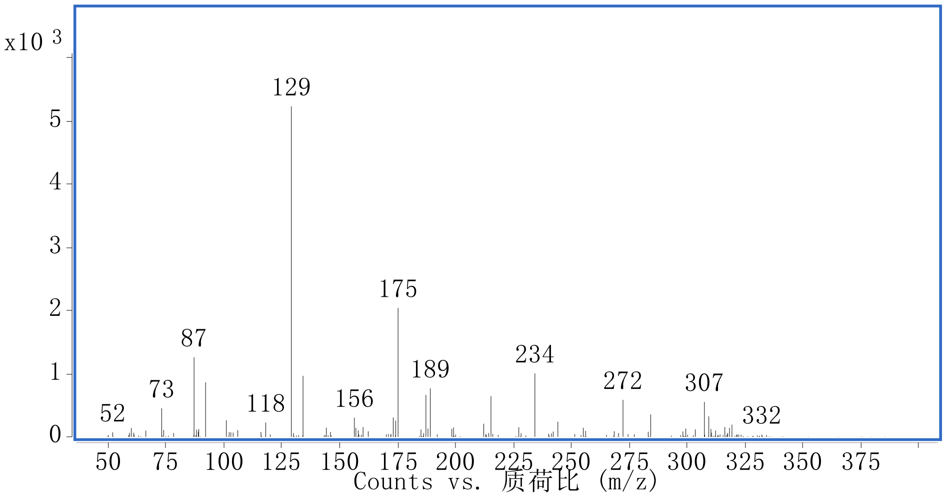

Supplementary Fig. S17 The mass spectra and proposed fragmentation 4,6-Me_2-D-Glc-2-N

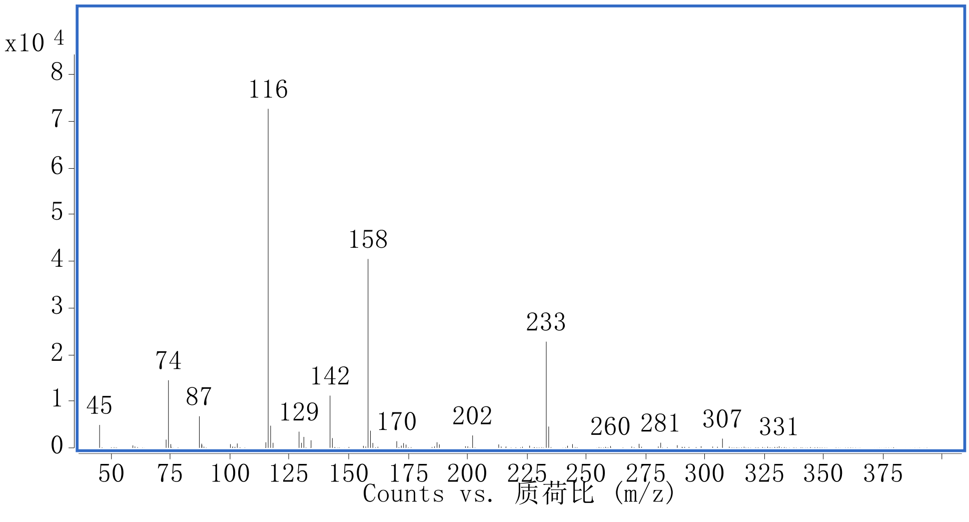

Supplementary Fig. S18 The mass spectra and proposed fragmentation 4-Me_1-D-Gal-2-N

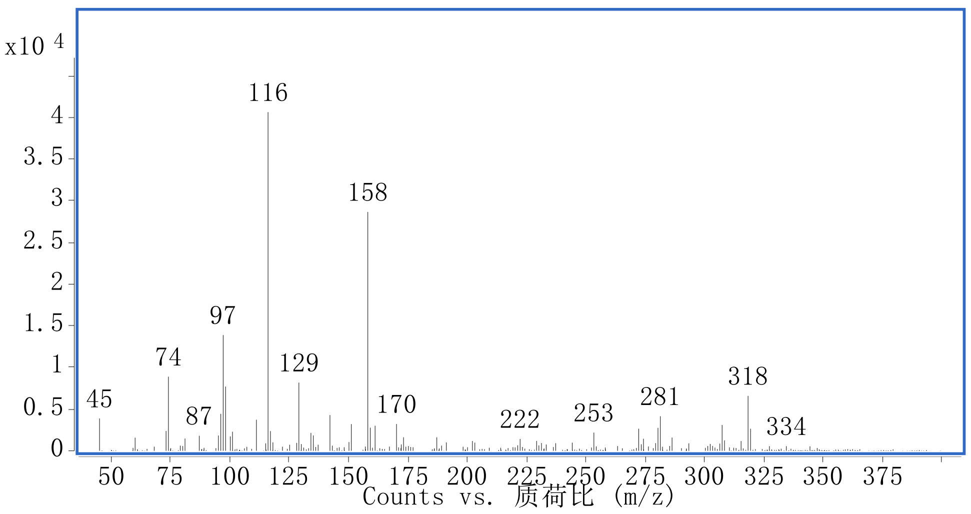

Supplementary Fig. S19 The mass spectra and proposed fragmentation 4-Me$_1$-D-Glc-2-N

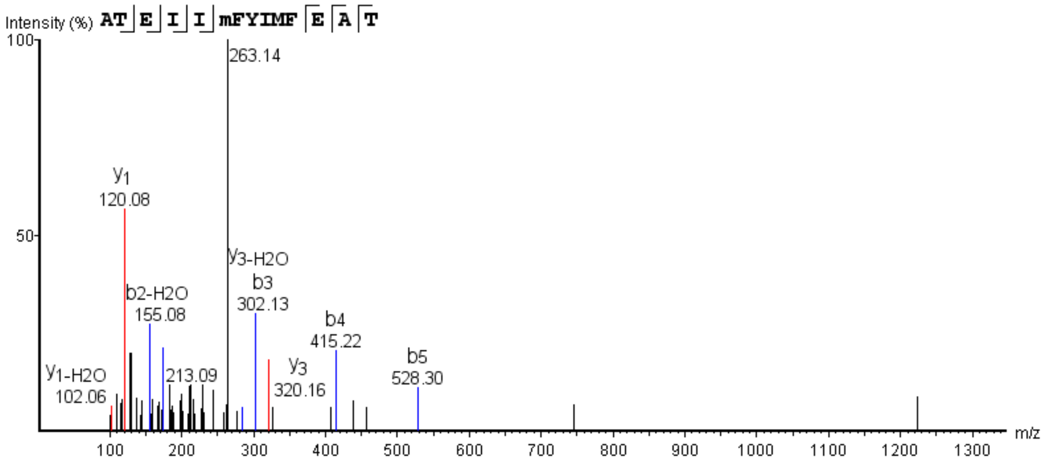

Supplementary Fig. S20 Two-stage mass spectrum of ATEImFYIMFIEAT peptide segment of T-ES

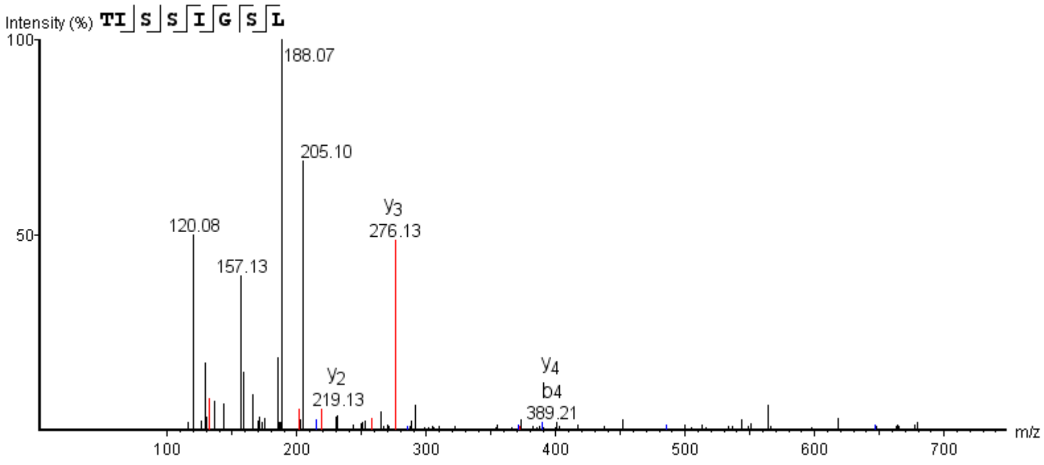

Supplementary Fig. S21 Two-stage mass spectrum of TISSIGSL peptide segment of T-ES

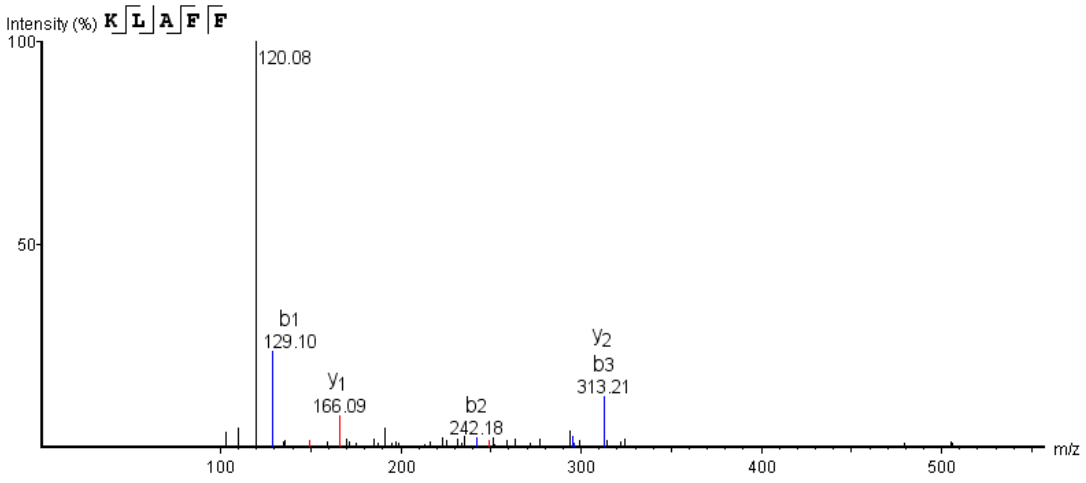

Supplementary Fig. S22 Two-stage mass spectrum of KLAFF peptide segment of T-ES

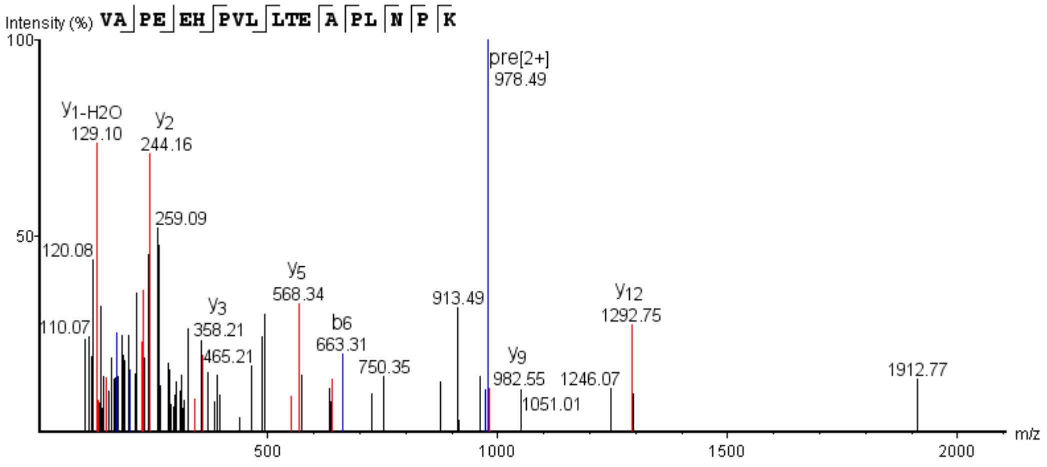

Supplementary Fig. S23 Two-stage mass spectrum of VAPEEHPVLLTEAPLNPK peptide segment of T-ES

Supplementary Table S1 Match peptides segment list information

No.	Peptide fragment	Molecular weight	m/z	Reliability	Area
1	ATEImFYIMFIEAT	789.4272	790.4354	45.16	9.13×10^5
2	TISSIGSL	776.4280	389.2180	35.34	3.34×10^5
3	KLAFF	624.3635	313.1895	38.07	1.06×10^5
4	ADNKSMI	777.3691	389.6900	18.94	2.37×10^7
5	VAPEEHPVLLTEAPLNPK	1953.0570	977.5369	36.87	7.23×10^4
